# Wiskott–Aldrich syndrome: Oral findings and microbiota in children and review of the literature

**DOI:** 10.1002/cre2.503

**Published:** 2022-02-24

**Authors:** Alessandra Lucchese, Sabina Cenciarelli, Maurizio Manuelli, Marta Marcolina, Federica Barzaghi, Valeria Calbi, Maddalena Migliavacca, Maria Ester Bernardo, Francesca Tucci, Vera Gallo, Federico Fraschetta, Silvia Darin, Miriam Casiraghi, Alessandro Aiuti, Francesca Ferrua, Maria Pia Cicalese

**Affiliations:** ^1^ Unit of Orthodontics, School of Dentistry Vita‐Salute San Raffaele University Milan Italy; ^2^ Unit of Orthodontics, Division of Dentistry IRCSS Ospedale San Raffaele Scientific Institute Milan Italy; ^3^ Unit of Dentistry, Research Center for Oral Pathology and Implantology IRCCS Ospedale San Raffaele Scientific Institute Milan Italy; ^4^ Pediatric Immunohematology and Bone Marrow Transplantation Unit, San Raffaele Scientific Institute Milan Italy; ^5^ Vita‐Salute San Raffaele University Milan Italy; ^6^ Private Practice, Milan, Bologna Pavia Italy; ^7^ San Raffaele Telethon Institute for Gene Therapy (SR‐Tiget), San Raffaele Scientific Institute Milan Italy

**Keywords:** microbiota, periodontitis, primary immunodeficiencies, Wiskott–Aldrich syndrome

## Abstract

**Objective:**

Wiskott–Aldrich syndrome (WAS) is a rare X‐linked primary immunodeficiency, characterized by micro‐thrombocytopenia, recurrent infections, and eczema. This study aims to describe common oral manifestations and evaluate oral microbioma of WAS patients.

**Material and methods:**

In this cohort study, 11 male WAS patients and 16 male healthy controls were evaluated in our Center between 2010 and 2018. Data about clinical history, oral examination, Gingival Index (GI) and Plaque Index (PI) were collected from both groups. Periodontal microbiological flora was evaluated on samples of the gingival sulcus.

**Results:**

WAS subjects presented with premature loss of deciduous and permanent teeth, inclusions, eruption disturbance, and significantly worse GI and PI. They also showed a trend toward a higher total bacterial load. *Fusobacterium nucleatum*, reported to contribute to periodontitis onset, was the most prevalent bacteria, together with *Porphyromonas gingivalis* and *Tannerella forsythia*.

**Conclusions:**

Our data suggest that WAS patients are at greater risk of alterations in the oral cavity. The statistically higher incidence of periodontitis and the trend to higher prevalence of potentially pathological bacterial species in our small cohort, that should be confirmed in future in a larger population, underline the importance of dentistry monitoring as part of the multidisciplinary management of WAS patients.

## 1 INTRODUCTION

Wiskott–Aldrich syndrome (WAS) is a rare X‐linked primary immunodeficiency, characterized by microthrombocytopenia, eczema, recurrent infections, and an increased incidence of autoimmunity and malignancies, with an incidence of ~1–10 per 1 million individuals (Aldrich et al., 1954; Ochs & Thrasher, 2006; Orange et al., 2004). The median age at diagnosis in patients without a family history is 24 months (Buchbinder et al., 2014). Advances in the diagnosis and the availability of definitive treatments have significantly improved the prognosis of WAS patients (Aiuti et al., 2013; Brigida et al., 2016; Ferrua et al., 2019; Marangoni et al., 2009; Moratto et al., 2011), while the morbidity and the quality of life of untreated patients is still poor (Massaad et al., 2013).

Both in pediatric and adult patients, WAS is typically associated with gingivitis and periodontitis; moreover, petechiae in the oral mucosa and active bleeding may also be observed (Kita et al., 2013; Lee et al., 2007; Reddy & Binnal, 2011; Salazar et al., 2013; Sollecito et al., 2005; Szczawinska‐Poplonyk et al., 2009). However, until now, few studies, mainly case reports, have addressed dental and oral manifestation in WAS patients, and the implications for oral health are yet to be clearly defined (Kita et al., 2013; Lee et al., 2007; Reddy & Binnal, 2011; Salazar et al., 2013).

The first aim of this study is to describe common oral signs and symptoms of individuals affected by WAS. The second aim is to evaluate the microbiota of the oral cavity in WAS patients, focusing mainly on the main pathogenic periodontal bacteria. The results emerging from these analyses may lead to early detection of oral disease in WAS patients, possibly helping to establish prevention measures to improve dental health in this patients' population.

## 2 PATIENTS AND METHODS

### 2.1 Patients

A total of 11 patients from an international cohort (all males, median age: 10 years, range 6–14), with WAS were evaluated at Pediatric Immunohematology and Bone Marrow Transplantation Unit of San Raffaele Hospital, Milan, Italy, between 2010 and 2018. At the time of evaluation, all patients were receiving substitutive intravenous immunoglobulins (IVIG), and antiviral, antifungal, and anti‐Pneumocystis prophylaxes. None of the patients had received definitive treatment with allogeneic hematopoietic stem‐progenitor cells transplantation (HSPC) or experimental gene therapy (GT) at time of analysis.

All patients presented with a severe Zhu score (> or =3), as a result of different mutations in WAS gene (Zhu et al., 1995). Four of these patients presented autoimmune/autoinflammatory manifestations (namely vasculitis, inflammatory bowel disease, pyoderma gangrenosum) and two were on treatment with immunosuppressants as steroids plus IL‐1RA and mesalazine, respectively (Brigida et al., 2016; Ferrua et al., 2019), at the time of oral sampling and microbiota evaluation. These four patients also were among the patients with the worst Zhu clinical score. None of the patients had assumed antibiotic therapy or prophylaxis in the 3 months prior to samples collection. A control group of 16 healthy male patients (median age: 10 years, range 8–12) was analyzed in parallel at the Unit of Dentistry, Division of Orthodontics of San Raffaele Hospital (Milan, Italy) between 2010 and 2018. Controls were matched for age and sex, and had to fulfill the following criteria: (1) no smoking; (2) no known systemic disease; (3) no alveolar bone loss visible on X‐ray; (4) no fixed restorations or removal dentures; (5) no use of antibiotics within 3 months before the study; (6) no periodontal therapy within the previous 6 months. Before the study, all participants received a standardized oral hygiene instruction by the same dental specialist and had a good oral hygiene profile.

Collection of biological specimen from patients was performed after parents/patients' signature of informed consent for biological samples collection, in the context of a protocol approved by the Ethical Committee of San Raffaele Hospital, Milan, Italy (project identification code Tiget06) on March 2015. All methods used in the study were non‐invasive. This study conformed to STROBE guidelines for observational human studies.

### 2.2 Methods

#### 2.2.1 Diagnostic examination

Clinical records were obtained, with particular focus on oral dental anamnesis for both groups. The routine oral and dental evaluation consisted of oral clinical examinations, intra and extra‐oral photographs, lateral cephalometric, and panoramic radiographs.

The following parameters were recorded from the clinical history (presence/absence): gingival bleeding, gingivitis, periodontitis, caries, oral abscesses, aphthous lesions, early deciduous teeth loss, early permanent teeth loss, permanent teeth inclusions, and eruption disturbances. We also excluded the presence of other factors that could alter oral flora, such as diabetes, gastroesophageal reflux, use of orthodontic devices (Lucchese et al., 2018).

Furthermore, Gingival Index (GI) and Plaque Index (PI) were used to measure bleeding and periodontal symptoms (Löe, 1967). Specifically, the GI was used for the assessment of the gingival condition and records changes in the gingiva. It scores the marginal and interproximal tissues separately on a scale from 0 to 3 (Löe, 1967). The scores of the four areas of the tooth were added and divided by four to give the GI for the tooth.

The PI is used together with GI, and in our study preceded the gingival examination (Löe, 1967). It is used on all the erupted teeth. There is no substitution for any missing tooth. It is used on all surfaces (mesial, occlusal, distal, lingual). This index measures the thickness of plaque on the gingival third. The PI of the patient was obtained by adding the values of each tooth and dividing by the number of teeth examined. The PI was scored for all teeth surfaces. Data collection was performed by a professional/skilled operator.

#### 2.2.2 Sample collection, DNA extraction, and quantification

After the removal of supra‐gingival biofilm, a sample of the gingival sulcus microbiota was taken from a single site using sterile paper probes to detect the presence of the main six periodontal bacteria (*Aggregatibacter actinomycetemcomitans*, *Porphyromonas gingivalis*, *Tannerella forsythia*, *Treponema denticola*, *Fusobacterium nucleatum*, *Campylobacter rectus*) (Bale et al., 2017; Lagier & Threadgill, 2014). The site of the analysis was the second superior left deciduous molar or the first–second superior left permanent premolar.

DNA was extracted from specimens after two consecutive incubations with lysozyme and proteinase K, in order to ensure an indiscriminate Gram+ and Gram− bacterial lysis. Once extracted, DNA was purified through a silica spincolumn (Sigma‐Aldrich, St. Louis, MO, United States). Quantitative polymerase chain reaction (PCR) of 16S rRNA genes was performed using the hydrolysis probes method to identify and evaluate the amount of the six aforementioned bacterial species (Human Oral Microbiome Database‐HOMD 16S rRNA RefSeq Version 10.1). The 845 sequences of 16S rRNA gene of the Human Oral Microbiome Database were aligned to find either a consensus sequence or less preserved spots, useful to optimize the specificity of primers and dual labeled hydrolysis probes. PCR oligonucleotide sequences were designed by Primer3web and Primer Express (Life Technologies) software. The specificity of PCR assays was also checked by Primer‐Blast. This technique consists in using dual‐labeled oligonucleotides, specific for a region of interest in the amplified target molecule. The hydrolysis probe is designed so that the length of the sequence places the 5′ fluorophore and the 3′ quencher in close enough proximity so as to suppress fluorescence, until the binding to the template; when extension reaches the bound hydrolysis probe, the 5′‐3′ exonuclease activity of the Taq DNA polymerase degrades the hydrolysis probe and the cleavage of the probe separates the fluorescent reporter molecule from the rest of the probe, allowing the reporter molecule to fluoresce (Scapoli et al., 2015).

#### 2.2.3 Methods error

The intraexaminer reproducibility was assessed after the examiner was trained according to the calibrated professional's criteria. Three healthy subjects of the control group were randomly selected and recruited for the calibration of GI and PI (Löe, 1967). The evaluation of Cohen's Kappa coefficient was used to assess intraexaminer agreement for the dichotomous variables, which have only two levels, that is, presence or absence of caries (Cohen, 1960; McHugh, 2012). Conversely, to quantify the measurement error of the variables, such as GI and PI, the formula described by Dahlberg was used (Dahlberg, 1940). Five patients were randomly selected and re‐observed by one operator; the scores (GI and PI) were reassigned twice by the same observer within 20 min of the first assessment (Dahlberg, 1940; Galvao et al., 2012). Intraexaminer error was <5% (95% confidence).

#### 2.2.4 Statistical analysis

Statistical descriptive analysis of the oral data and of medical signs and symptoms recorded was performed using IBM SPSS Statistics 21 software. The statistical analysis of microbiological characterization was performed on relative quantity calculated as ratios between the amount of each bacterial species (absolute quantity) and the total quantity of the bacteria. This allowed the reduction of variability due to random factors such as specimen preservation, DNA extraction efficiency and purification yield, as well as variability due to systematic factors (i.e., the higher amount of bacteria expected in specimens from deeper pockets characterizing oral inflammation) (Scapoli et al., 2015). To compare the different general health conditions between the two groups, the Fisher's exact test was applied; two‐tailed *t* test was applied to compare clinical parameters and bacterial species in healthy controls and WAS patients. Pearson correlation was used to measure the correlation between Zhu score and GI or PI.

## 3 RESULTS

### 3.1 General health status

**Figure 1:**
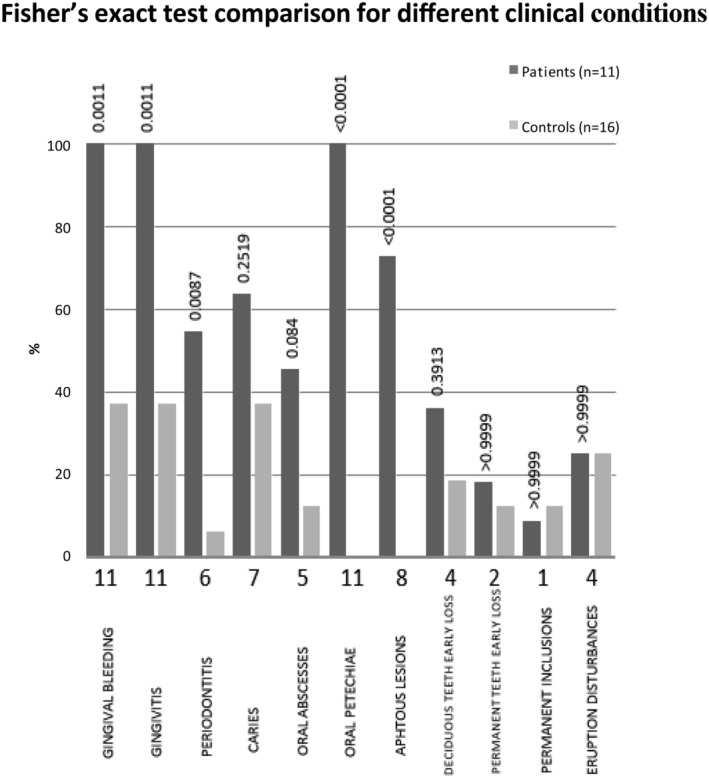
Wiskott–Aldrich syndrome (WAS) patients versus healthy controls: Fisher's exact test comparison for different clinical conditions. Oral findings in WAS patients. The gray boxes highlight the significant differences (*p* value <0.05) between the WAS and healthy controls populations. The absolute number of patients of the cohort with specific manifestation is reported under the graph (total patients *N* = 11)

All the patients analyzed presented with classical WAS, characterized by a history of recurrent infections, eczema, thrombocytopenia, and hemorrhagic diathesis (associated to major bleeding events in the 54.6% of patients), showing a health‐related quality of life (HRQOL) of 75.0 ± 10.0 (Selewski et al., 2015). In addition, 63.6% of these patients presented with anemia (Figure 1). These disease‐specific clinical features were confirmed to be significantly different in WAS patients as compared to the control group of healthy patients (Figure 1).

### 3.2 Oral health status

**Figure 2:**
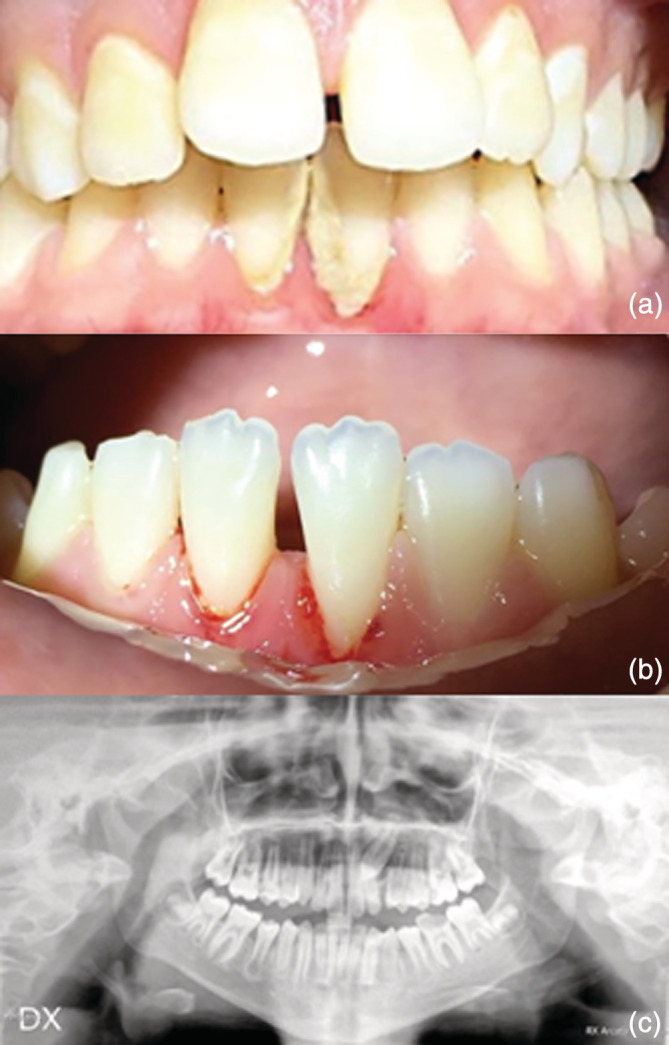
Intraoral examination revealed a severe oral infection associated with gingival bleeding, recession, and periodontitis (a, b). Panoramic X‐rays show the inclusion of the maxillary left permanent canine and a destructive carious lesion involving the mandibular left permanent first molar (c)

The major oral findings detected in WAS patients were: oral petechiae, gingivitis, and gingival bleeding in all of them, followed by aphthous lesions (72.7%), periodontitis (54.6%), and severe oral infections such as caries (63.6%) and oral abscesses (45.5%) (Figure 1). No other enamel abnormalities were detected. In comparison with age matched healthy subjects, the incidence of gingival bleeding, gingivitis, periodontitis, oral petechiae, and aphthous lesions was significantly higher in WAS patients (Figures 1 and 2a,b). Regarding orthodontic malocclusions in WAS patients, oral examination, and orthopantomographies revealed premature loss of deciduous (36.36%) and permanent (18.18%) teeth, inclusions (9.1%), and eruption disturbance (36.36%) (Figures 1 and 2c), even if no major differences were detected once compared to healthy subjects (Figure 1). Splanchnocranial skeletal alterations were not observed in WAS patients.

GI (mean value = 1.66, standard deviation = 0.63) and PI (mean value = 1.53, standard deviation = 0.73) showed moderate periodontal inflammation and moderate presence of plaque in WAS patients, as shown in Table 1. GI and PI resulted worse in patients as compared to controls (Table 1). GI and PI were more severe in some of the patients with a higher Zhu score, despite the lack of a statistically significant correlation (*p* value 0.47 and 0.92, respectively).

**Table 1:** Clinical parameters (PI, GI) in patients with Wiskott–Aldrich syndrome and in controls

	PI	GI
*WAS*		
Mean	1.53	1.66
Min	0.38	0.86
Max	2.74	2.65
Standard deviation	0.73	0.63
Median	1.61	1.73
*Controls*		
Mean	0.82	0.75
Min	0.22	0.1
Max	2.15	2.22
Standard deviation	0.48	0.56
Median	0.73	0.55
Two tailed *t* test *p* value (CTR–WAS)	0.108	0.0007
Difference between means ± SEM (CTR–WAS)	−0.6489 ± 0.2356	−0.9041 ± 0.2338

Abbreviations: GI, Gingival Index; PI, Plaque Index; WAS, Wiskott–Aldrich syndrome; CTR, Control.

### 3.3 Oral microbiota

The absolute and the relative amount of each analyzed bacterium were retrieved in the samples of the 11 WAS patients and in the control group, in addition to the total charge of crevicular bacteria.

Despite the limitation given by the small sample size, due to the rarity of the disease, the oral microbiota samples revealed an almost 30% higher bacterial load in WAS patients, including species strongly associated with periodontitis (Bale et al., 2017; Lagier & Threadgill, 2014), if compared to age matched healthy donors. In particular, we detected a high to very high bacterial load for *P. gingivalis* (bacterial load: absolute amount 629, relative amount 0.85%), and *T. forsythia* (bacterial load: absolute amount 650, relative amount 0.82%). *F. nucleatum*, reported to contribute to the onset of periodontitis, was the most prevalent bacteria detected, with a very high bacterial load (bacterial load: absolute amount 1484, relative amount 1.88%) (Table 2) (Hartsfield, 1994).

**Table 2:** Distribution of the main bacteria in oral cavity in WAS patients and compared with healthy controls

	*Aggregatibacter actinomycetemcomitans*	*Porphyromonas gingivalis*	*Tannerella forsythia*	*Treponema denticola*	*Fusobacterium nucleatum*	*Campylobacter rectus*	Total charge
Absolute amount							
Mean	65	629	651	32	1484	590	89,287
Min	0	0	0	0	0	18	34,758
Max	331	1802	1404	156	3246	1742	152,984
Standard deviation	110	643	542	54	862	587	38,960
Median	0	732	829	0	1623	327	92,718
Relative amount (%)							
Mean	0.06	0.85	0.82	0.05	1.88	0.64	100.00
Min	0.00	0.00	0.00	0.00	0.00	0.02	100.00
Max	0.27	2.87	1.82	0.28	3.33	1.54	100.00
Standard deviation	0.10	0.99	0.70	0.09	1.12	0.54	0.00
Median	0.00	0.48	0.86	0.00	1.43	0.75	100.00
Two tailed *t* test *p* value (CTR–WAS)	0.9308	0.5515	0.4045	0.5506	0.0967	0.5204	0.0576
Difference between means ± SEM (CTR–WAS)	−0.002568 ± 0.02928	−0.1765 ± 0.2923	−0.1861 ± 0.2195	0.02045 ± 0.03381	−0.6387 ± 0.3700	−0.1128 ± 0.1730	−27,413 ± 13,772

*Note*: Absolute amount: <100 is equal to “moderate” bacterial load; <500 means “high” bacterial load and >500 corresponds to “very high” bacterial load. Relative amount: <0.1000 is equal to “moderate” relative charge; <0.5000 is equal to “high” relative charge; <1.0000 corresponds to “very high” relative charge.

Abbreviation: WAS, Wiskott–Aldrich syndrome; CTR, Control.

In WAS patients, a trend toward a higher total bacterial load in comparison to the control group was observed (*p* = 0.0576), while no significant differences were seen between the bacterial species analyzed in the two groups (Table 2).

## 4 DISCUSSION

This study aims to describe the oral, mucosal, and dental status of a cohort of 11 patients with WAS and to characterize their microbiological periodontal flora comparing them with age‐matched healthy subjects. To our knowledge, this represents the first study to investigate oral manifestations in a relatively large series of WAS patients, having being published only case reports about few WAS patients (Table 3) (Boraz, 1989; Kita et al., 2013; Lee et al., 2007; Porter et al., 1994; Reddy & Binnal, 2011; Salazar et al., 2013). In the eight cases described, gingival bleeding and gingivitis were reported in all patients (Boraz, 1989; Kita et al., 2013; Lee et al., 2007; Porter et al., 1994; Reddy & Binnal, 2011; Salazar et al., 2013). Moreover, pain and swelling of the gingiva (Kita et al., 2013; Reddy & Binnal, 2011), cheilitis (Lee et al., 2007), oral petechiae (Boraz, 1989; Lee et al., 2007; Porter et al., 1994; Reddy & Binnal, 2011), oral infections (Kita et al., 2013; Lee et al., 2007; Reddy & Binnal, 2011), dental caries (Kita et al., 2013; Lee et al., 2007; Reddy & Binnal, 2011), oral abscesses, and radicular cyst (Kita et al., 2013; Reddy & Binnal, 2011), and premature loss of the dental elements have also been observed, with necessity of pulpotomy in some patients (Kita et al., 2013; Lee et al., 2007; Reddy & Binnal, 2011; Salazar et al., 2013). Importantly, oral findings appear more common in WAS patients with a severe clinical score or in patients on supportive treatments (Boraz, 1989; Ferrua et al., 2019; Kita et al., 2013; Lee et al., 2007; Porter et al., 1994; Reddy & Binnal, 2011; Salazar et al., 2013). While thrombocytopenia and platelet dysfunction favor gingival bleeding (Boraz, 1989; Ferrua et al., 2019; Kita et al., 2013; Lee et al., 2007; Porter et al., 1994; Reddy & Binnal, 2011; Salazar et al., 2013; Sereni et al., 2019), gingivitis, and oral petechiae (Boraz, 1989; Ferrua et al., 2019; Lee et al., 2007; Porter et al., 1994; Reddy & Binnal, 2011; Sereni et al., 2019), the underlying immunodeficiency may facilitate the occurrence of oral infections (Kita et al., 2013; Lee et al., 2007; Reddy & Binnal, 2011; Salazar et al., 2013). Furthermore, periodontitis and infections can rely on gingival bleeding: indeed, thrombocytopenia can trigger periodontitis through a continuity lesion of the mucosa, allowing the entry of bacteria and facilitating their proliferation in the tissues. On the other side, periodontitis causes an increase of mucosal fragility, making it more prone to gingival bleeding. On the other hand, medical and surgical interventions in the oral cavity in WAS patients may require a more accurate management due to the bleeding issues related to low platelet count (Ferrua et al., 2019; Salazar et al., 2013).

**Table 3:** Review of the literature

Author/year	Boraz, 1989	Porter et al., 1994	Lee et al., 2007	Reddy & Binnal, 2011	Salazar et al., 2013	Kita et al., 2013
Study design	Case report	Case report	Case report	Case report	Case report	Case report
Patients	1	1	2	2	1	1
Age	n.r.	n.r.	Patient 1: 2 years 11 months Patient 2: 2 years 6 months	Patient 1: 6 years Patient 2: 4 years	Patient 1: 6 years	Patient 1: 14 years
Collection methods	OE	OE	OE, Periapical RX	OE	OE	OE, Ortopantomography

Results: Oral findings at T0 (before dental therapies)	Patients (*N*)	Patients (*N*)	Patients (*N*)	Patients (*N*)	Patients (*N*)	Patients (*N*)
Gingival bleeding	1	1	2	2	1	1
Gingivitis	1	1	2	2	1	1
Periodontitis	‐	‐	‐	‐	‐	‐
Caries	‐	‐	1	1	‐	1
Oral abscesses	‐	‐	1	1	‐	1
Oral petechiae	1	1	2	2	1	‐
Aphthous lesions	1	1	1	2	‐	‐
Deciduous teeth early loss	‐	‐	1	‐	1	1
Permanent teeth early loss	‐	‐	‐	‐	‐	1
Permanent teeth inclusions	‐	‐	‐	‐	‐	1
Eruption disturbances	‐	‐	1	‐	1	1

Abbreviations: n.r., not reported; OE, oral examination; RX, radiograph.

Our study has shown that an increase in the main periodontal parameters (GI and PI) is observed in patients with WAS, highlighting that in these patients there may be a greater risk of oral disease. In particular, GI alteration confirms that inflammation is a main issue in WAS patients and could affect the oral cavity as well as other tissues (i.e., skin, other parts of gastrointestinal tract, kidneys), as reported, in particular patients with autoimmune/autoinflammatory clinical manifestations (Ferrua et al., 2019). Existing oral inflammatory conditions could be exacerbated in WAS patients, resulting in further loss of supporting structures. Moreover, in case orthodontic treatment is required, it may be delayed or altered in WAS children if the periodontal tissues are not healthy, and fixed appliances may further increase the gingival bleeding due to low platelet count.

Despite the small sample size, due to the rarity of WAS, and the consequent absence of a strong statistical evidence, our study highlighted a trend of WAS patients to an increase in bacterial load and in severity of main periodontal parameters (GI and PI) versus healthy controls, resulting in a greater risk of oral disease. In particular, WAS patients seemed to show a higher load of *P. gingivalis* and *T. forsythia*, a prototype polybacterial pathogenic consortium in periodontitis, and *F. nucleatum*, as compared to controls. All these bacterial species are prominent components of the oral microbiota and a common colonizers of the oral epithelium, but they may become highly destructive and proliferate in periodontal lesions in presence of host predisposing factors (i.e., impaired mucosal immunity, local inflammation), contributing to the development of periodontal disease. Furthermore, while the association between these bacteria and periodontal disease has long been reported, the role of oral flora in systemic diseases is an object of recent studies. For example, *F. nucleatum* has recently been implicated in colorectal cancer and *P. gingivalis* and *T. forsythia* may be implied in atherosclerosis, chronic lung disease, rheumatoid arthritis, and gastrointestinal neoplasms (i.e., colon and esophagus) (Fiorillo et al., 2019; Hashemi Goradel et al., 2019; Malinowski et al., 2019). This may impact even more dramatically in patients with primary immunodeficiencies and should be carefully monitored. It will be interesting to conduct further studies to evaluate the specific genomics of these species in WAS patients in order to define their metabolism, their ability to colonize epithelial cells and their influence upon the host immunity and the variability of disease phenotype (i.e., presence of autoimmunity/autoinflammation, food allergy, infections' susceptibility, etc…).

Despite the knowledge collected in the last few decades, many questions about oral pathophysiology in WAS patients remain unanswered. Hartsfield proposed WAS in itself as a cause of premature exfoliation of deciduous teeth and argued that the immunodeficiency could result also in early loss of permanent teeth (Hartsfield, 1994). To date, it is still unknown if these manifestations in WAS patients could be related to WASP defect in the osteoclasts compartment, that might determine their altered cellular function. Indeed, WASP‐deficient mice presents defects in bone reabsorption due to the critical role of WASP in actin reorganization and podosome formation (Calle et al., 2004; Chellaiah et al., 2007). On the other hand, these manifestations are likely to be related to untreated inflammatory and infectious processes, which can give rise to early loss of deciduous teeth, delay in the eruption of the permanent teeth, disruption in the physiological sequence of teeth eruption and malocclusions, leading to further orthodontic problems.

It is widely reported that oral infections seem to increase the risk of cardiovascular disease and pulmonary disease (Bale et al., 2017; Li et al., 2000). The impact could be even higher in case of a primary immunodeficiency, therefore a more accurate prevention and treatment plan of the oral deterioration is mandatory in WAS patients to reduce local inflammation, bacteremia, and to prevent systemic complications.

It is known that orthodontic treatments can cause a higher amount of dental plaque and a higher risk of enamel and dentin alterations, with the possible appearance of white spot lesions and dental caries (Lucchese et al., 2018; Prati et al., 1999; Lucchese et al., 2011), even in healthy subjects. Again, these corrective measures could be even more critical in WAS patients, who are more susceptible to dental infections.

Finally, to date there are no reports regarding the outcome of surgical procedures, as orthopedic, mid‐palatal suture expansion, and orthodontic therapies in WAS patients and their appropriateness in this disease needs to be established. These could be burdened by more severe complications, considering their pro‐inflammatory genetic susceptibility and the potential impact of the underlying immune defect on bone homeostasis (Raggatt & Partridge, 2010).

In conclusion, orofacial manifestations of WAS are part of the wide spectrum of this disease, and a few reports are currently present in published literature. Early diagnosis and oral examination could result in a significant improvement of the general and oral health in WAS subjects and could help to prevent additional complications, such as recurrent infections or the premature loss of deciduous and permanent teeth and subsequent orthodontic alterations. Therefore, it is mandatory to increase awareness of this entity and to include a dental specialist evaluation in the multidisciplinary management of these patients in order to specifically address oral cavity alterations.

This is the first report addressing findings of oral manifestations and microbiota in a cohort of 11 WAS patients, with comparison to healthy subjects. Future studies on a larger WAS population are needed to add new details and to go deeper in the understanding of the factors that may underlie the pathogenesis of oral manifestations in WAS patients. We also think that the extension of the sampling to patients' family members in a prospective fashion and the study of a larger number of oral species could be interesting. Moreover, it will be interesting to observe how these findings may change after definitive treatments as HSCT or GT.

## CONFLICT OF INTEREST

The authors declare no conflicts of interests.

## AUTHOR CONTRIBUTIONS

Alessandra Lucchese contributed to the conception, data acquisition, data interpretation, and wrote the manuscript. Sabina Cenciarelli contributed to the writing of the paper, data acquisition and data analysis. Maurizio Manuelli contributed to data interpretation and critically revised the manuscript. Maurizio Manuelli, Federica Barzaghi, Valeria Calbi, Maddalena Migliavacca, Sabina Cenciarelli, Maria Ester Bernardo, Francesca Tucci, Vera Gallo, Federico Fraschetta, Silvia Darin, and Miriam Casiraghi contributed to data acquisition and critically revised the manuscript. Alessandro Aiuti contributed to conception and critically revised the manuscript. Francesca Ferrua and Maria Pia Cicalese contributed to conception, design, data acquisition, analysis, and interpretation, and critically revised the manuscript. All authors gave final approval and agree to be accountable for all aspects of the work.

## Data Availability

The data that support the findings of this study are available from the corresponding author upon reasonable request.
